# Functional knee positioning in patients with valgus deformity undergoing image-based robotic total knee arthroplasty: Surgical technique

**DOI:** 10.1051/sicotj/2025001

**Published:** 2025-02-10

**Authors:** Pietro Gregori, Christos Koutserimpas, Andrea De Fazio, Sarah Descombris, Elvire Servien, Cécile Batailler, Sébastien Lustig

**Affiliations:** 1 Orthopaedics Surgery and Sports Medicine Department, FIFA Medical Center of Excellence, Croix Rousse Hospital, Hospices Civils de Lyon, Lyon North University Hospital 103 Grande Rue de la Croix Rousse 69004 Lyon France; 2 Fondazione Policlinico Universitario Campus Bio-Medico Via Alvaro del Portillo, 200 00128 Roma Italy; 3 LIBM-EA 7424, Interuniversity Laboratory of Biology of Mobility, Claude Bernard Lyon 1 University 43 Bd du 11 Novembre 1918 69100 Villeurbanne, Lyon France; 4 Univ Lyon, Claude Bernard Lyon 1 University, IFSTTAR, LBMC UMR_T9406 69622 Lyon France

**Keywords:** Personalized knee arthroplasty, Robotic knee, Functional alignment, Knee anatomy, Valgus

## Abstract

*Background*: Functional knee positioning (FKP) represents an innovative personalized approach for total knee arthroplasty (TKA) that reconstructs a three-dimensional alignment based on the optimal balance of soft tissue and bony structures, but it has mostly been described for varus knee deformity. *Surgical technique*: Valgus deformities present specific challenges due to altered bone remodeling and soft tissue imbalances. Using robotic assistance, FKP enables precise intraoperative assessment and correction of compartmental gaps, accommodating each individual’s unique anatomy and laxities. The distal femoral cut is calibrated for 9 mm resection at the intact medial femoral condyle and adjusted on the lateral side to accommodate bone wear, while the tibial plateau resection aims for 8 mm from the medial side and 4–6 mm from the lateral side. Intraoperative evaluations of mediolateral laxities are performed at extension and 90° flexion. Adjustments are made to femoral and tibial cuts to balance gaps, aiming for 0 mm in posterior stabilized implants and minimal discrepancies in cruciate-retaining designs with lateral gap looser in flexion. *Discussion*: FKP emphasizes soft tissue-driven adjustments with the use of robotic platforms. Hence, intact soft tissue envelope of the knee is essential. This technique holds significant promise for managing valgus deformities in TKA, but further research is needed to evaluate its functional outcomes.

## Introduction

The development of alignment strategies for total knee arthroplasty (TKA) over the past 20 years has focused on tailoring implant placement to the patient’s specific anatomy to achieve better clinical and functional outcomes [1]. Although mechanical alignment (MA) has provided a reliable and reproducible method, it has many limitations in restoring the anatomic-specific characteristics of each patient, since only 50% of the population has a neutral mechanical axis [2]. The emergence of more individualized alignment philosophies is also related to the fact that many patients may complain about limited functional results in the post-surgical period [2]. In recent years, numerous alternative alignment techniques have been proposed to improve functional outcomes and restore native knee kinematics such as kinematic alignment (KA), inverse kinematic alignment (iKA), restricted kinematic alignment (rKA) [3]. Among these, functional knee positioning (FKP) represents a distinct approach. The goal of FKP is the reconstruction of a three-dimensional alignment based on an optimal balance of soft tissue and bony structures with the use of robotics [4]. Robotic tools enable the assessment and correction of inter-compartmental gaps, allowing for the intraoperative accommodation of each individual’s unique laxities and anatomy with minimal overall complications and drawbacks [5]. A valgus knee deformity presents a considerable challenge in TKA due to its impact on bone remodeling and the contraction or lengthening of soft tissues [6].

This surgical technique article aimed to meticulously illustrate the FKP technique for the valgus morphotype, with the use of an image-based robotic platform for preoperative and intraoperative planning (Mako, Stryker, Mako Surgical Corp., Fort Lauderdale, FL, USA).

## Surgical technique

The patient is placed in a standard supine position, with one arm laying on a lateral support in abduction, and the other one on the surgical table. A lateral pad centered on the thigh and one distal pad to keep the knee at 90° intraoperative are positioned (Video 1).

### Step 1: Preoperative planning for distal femoral and tibial plateau resection

**Figure 1:**
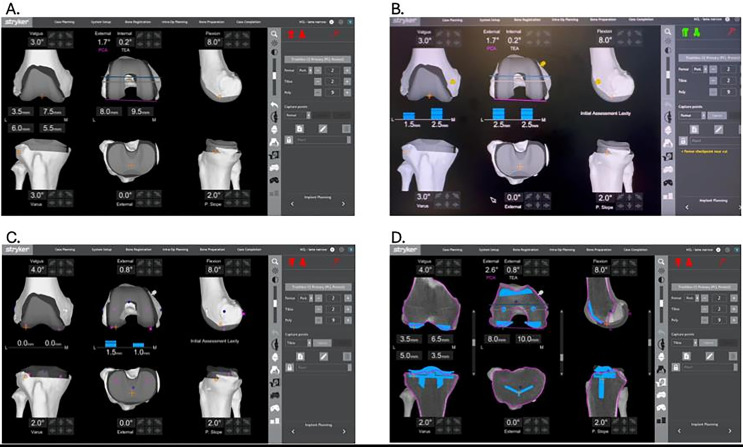
An example of an intra-operative functional alignment planification in a right knee. (A) Pre-operative planning for implant positioning and thickness of the robotic bone cuts. (B) Gaps existing between the preoperative planning and the intra-operative assessment of the knee laxities. (C) Residual Gaps after the functional gap-balancing of the knee. (D) Final implant positioning and thickness of the robotic cuts.

Computer navigation (Orthomap ASM, Stryker, Michigan, USA) is used to ensure the functional positioning of the implants in the three-dimensional planes [6]. The preoperative plan aims to resurface the knee while preserving each patient’s unique anatomy, whenever possible. The distal femoral resection is calibrated for 9 mm resection at the intact medial femoral condyle (7 mm of bone plus 2 mm of cartilage) and 4–6 mm from the lateral femoral condyle to account for any bone wear [7]. The tibial plateau resection targets 8 mm from the medial side (6 mm bone plus 2 mm cartilage) and 4–6 mm on the lateral side if bone wear is present. The planned combined medial resection should match the thickness of the implant with the thinnest tibial liner at 17 mm. Adjustments are made if the implant’s trochlea orientation does not restore the patient’s anatomy or if compartmental thickness differences exceed 1.5 mm (Figure 1).

### Step 2: Approach and pins’ placement

**Figure 2:**
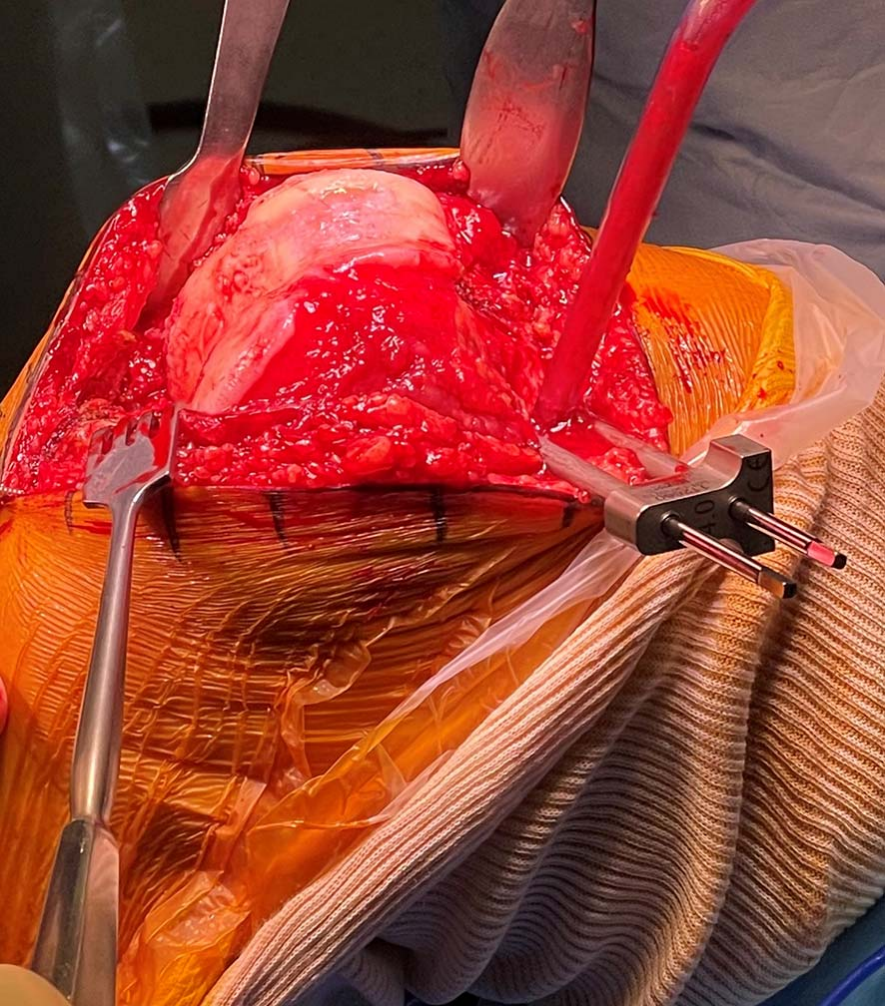
Femoral pins are positioned anteromedially on the medial condyle.

**Figure 3:**
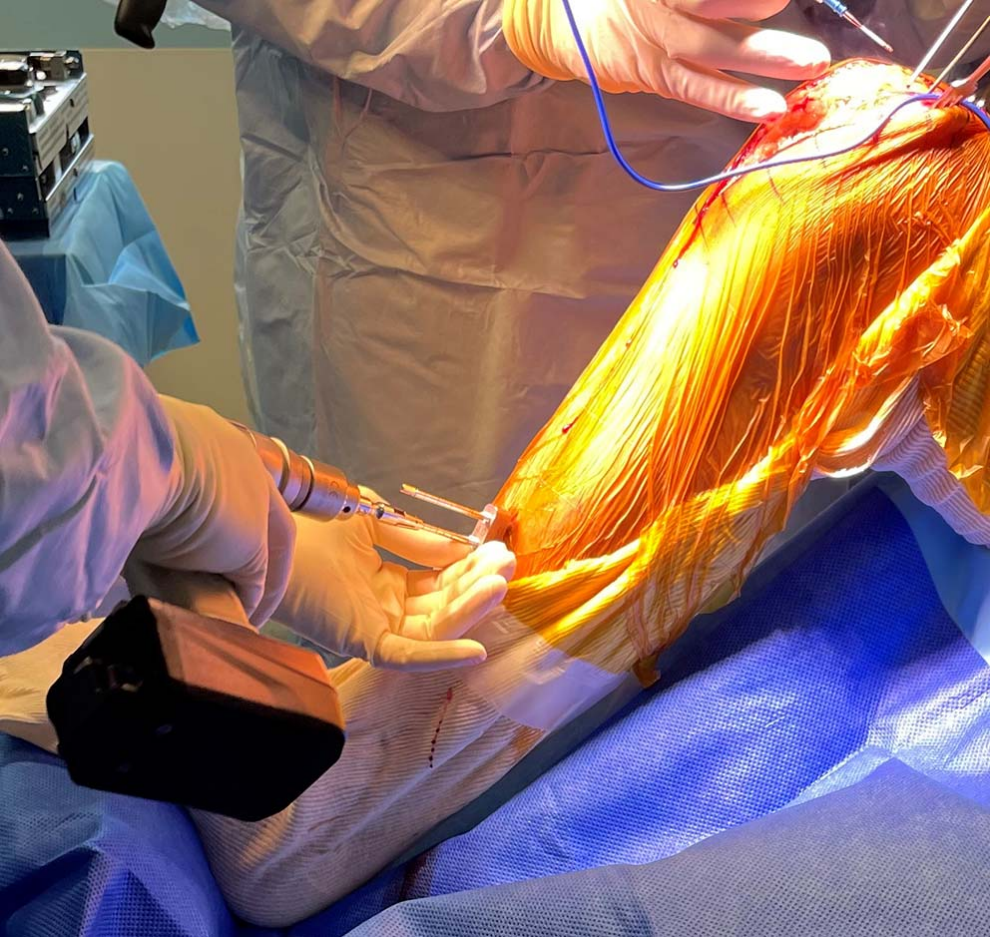
Tibial pins are placed through two anteromedial stab incisions on the distal diaphysis.

**Figure 4:**
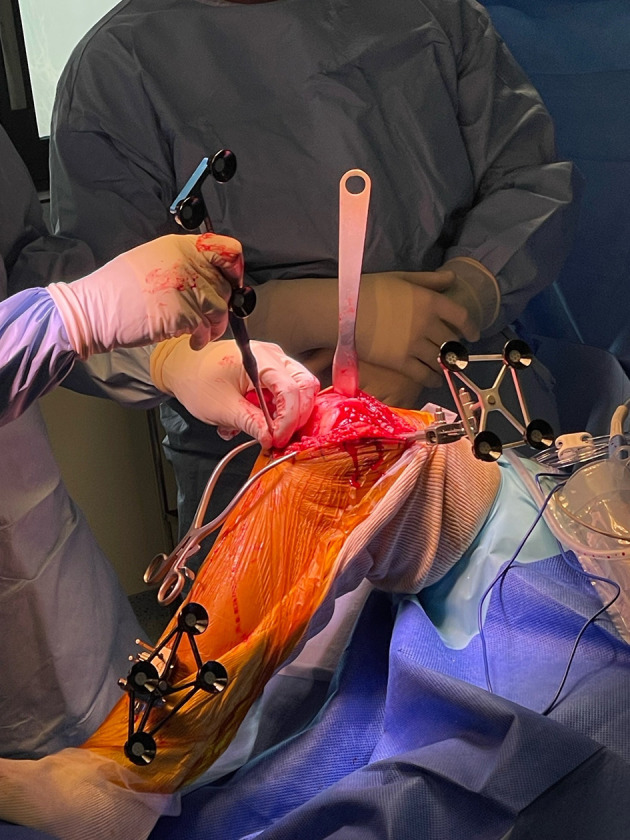
Arrays positioned. The reference points are registered to match the anatomy of the patient to the preoperative CT scan.

A medial subvastus or a medial parapatellar approach with a central incision is preferably used. After accessing the joint, femoral pins are positioned anteromedially on the medial condyle, while tibial pins are placed through two anteromedial stab incisions on the distal diaphysis. Intraincisional pins positioning is also an alternative option. Arrays are positioned and reference points are registered to match the anatomy of the patient to the CT scan (Figures 2–4).

### Step 3: Intraoperative alignment assessments and evaluation of the laxities

Intraoperative coronal alignment assessments are collected at full extension, 90° flexion, and maximum flexion, followed by an evaluation of the laxities in the medial and lateral compartments.

Laxities are evaluated at approximately 10° flexion, to avoid posterior capsule tension, and at 90° of flexion in both compartments, using manual valgus and varus stress in extension and specific spoons in flexion. Intraoperative evaluations allow real-time comparison between the preoperative plan cuts and the observed laxities of each compartment (Figure 1).

### Step 4: Femoral and tibial cuts adjustment

To optimize knee balance, femoral and tibial cuts are adjusted to achieve 0 mm gaps in extension and flexion for the lateral and medial compartments in posterior stabilized (PS) implants, while for cruciate retaining (CR) implants, up to 1.5 mm and 1 mm gaps in flexion for the lateral and medial compartments respectively are aimed. The thickness of the cuts is kept between 1.5 mm and 10 mm. Finally, a strict evaluation of the trochlear compatibility of the implant with the native anatomy and the tibial implant coverage is performed.

### Step 5: Femoral, tibial cuts and implant of the trial components

Robotic guidance ensures precise femoral and tibial cuts with the use of the robotic arm. Trial components are then inserted to verify the femoral medio-lateral positioning, tibial rotation, and the reliability of the robotic cuts.

### Step 6: Intraoperative check of the alignment and gaps

The final coronal alignment is re-assessed at full extension, 90° flexion, and maximum flexion, targeting preferably a “safe zone” of 177°–183°. Residual gaps and patellar tracking are evaluated, while the tibial rotation is determined by a combination of the floating method, the Akagi’s line, and the robotic matching with the preoperative plan (Video 1).

### Step 7: Placement of the final implant

**Figure 5:**
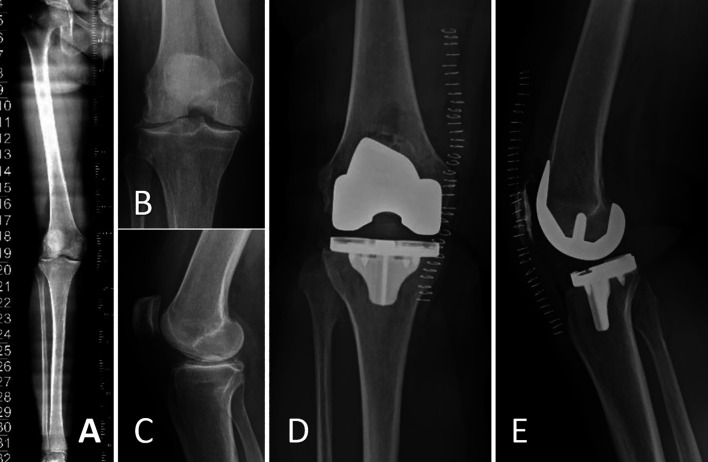
Pre- and post-operative X-ray views of a valgus knee with osteoarthritis treated with image-based total knee arthroplasty under the principles of Functional knee positioning. (A) Preoperative long axis weight-bearing Anteroposterior (AP) view, (B) AP view of the knee, (C) Lateral view of the knee, (D and E) AP and lateral postoperative X-ray views showing the implantation of a cementless femoral and tibial component (Triathlon, Stryker, MI, USA).

The final implants, either CR or PS fixed bearing designs (Triathlon, Stryker, MI, USA), are then positioned and the final checking may be performed (Video 1 and Figure 5).

## Discussion

The aim of this article was to describe and illustrate the technique of FKP for the valgus morphotype. TKA for the valgus knee has been considered more challenging compared to TKA for varus deformity [6], largely due to the presence of multiple phenotypes associated with the underlying origin of the deformity [8]. The complexity of this scenario may require a more individualized approach. Studies on other personalized alignments in TKA for the valgus knees have shown the necessity of frequent ligament releases to obtain an acceptable result [9].

Considering that there are still many surgeons who prefer to use MA for valgus knees; it is important to thoroughly describe the FKP in this deformity. FKP could be a solid option for these cases, since it represents a soft tissue-driven personalized alignment. Nevertheless, it should be noted that careful patient selection is of utmost importance. For patients to be able to undergo FKP the soft tissue envelope of the knee should be intact. Hence, patients with rheumatoid arthritis, previous traumas, ligamentous injuries, and high tibial osteotomies do not represent optimal candidates for FKP. Furthermore, FKP focuses on the anterior compartment of TKA by carefully examining the patellofemoral joint which could also play an important role in overall outcomes [10].

The aim of FKP is to restore the patient’s constitutional alignment, corrected by the accommodation of each patient’s individual laxities and anatomy [4]. This approach permits the evolution of a three-dimensional alignment based on an optimal balance of soft tissue and bony structures, achieved through the innovative and efficacious use of robotic platforms. For the implementation of FKP, the use of robotics is essential. TKA with the use of robotics has been proven to be a safe and reliable method, while many systems are now available [5, 11].

Over the past decade, personalized alignment techniques for TKA have gained significant attention for their potential to optimize outcomes by tailoring alignment to individual patient’s anatomy and biomechanics [12, 13]. Techniques such as KA, rKA, iKA, and functional alignment or FKP have emerged as alternatives to traditional MA [1]. KA aims to restore the patient’s native knee kinematics by aligning components to the pre-arthritic anatomy, which has shown improved patient satisfaction and functional outcomes in certain cohorts. rKA modifies this approach by introducing minor constraints to ensure mechanical safety and durability, balancing kinematic restoration with implant longevity [14]. iKA inverts the kinematic philosophy for specific cases, seeking to optimize alignment by adapting the femoral positioning to the tibial orientation of the patient. FKP, which uses intraoperative tools or sensors to achieve alignment based on real-time soft tissue balance measured through laxities, has demonstrated promising early outcomes in enhancing knee function and reducing pain [15]. Collectively, these techniques highlight the shift towards a more patient-centric approach in TKA, with studies showing improved functional scores, higher satisfaction rates, and more natural knee kinematics. However, the long-term durability of these approaches remains under investigation, warranting further high-quality research.

FKP holds significant promise for the future. This article thoroughly exhibited the implementation of the personalized alignment technique in patients with valgus deformity, undergoing image-based robotic TKA. Future studies evaluating the outcomes of FKP in the setting of valgus deformity are essential.

## Conclusion

This surgical technique article described and illustrated the technique of FKP for managing the valgus morphotype using the image-based robotic system. The technique is effective and reproducible. Careful patient selection is of utmost importance, since the soft-tissue envelope of the knee should be intact. It is evident that further studies are necessary to assess the short- and long-term clinical and functional outcomes.

## Data Availability

Data is available upon reasonable request to the corresponding author.

## Supplementary material

**Video 1:** Functional knee positioning in a valgus knee with the subvastus approach and intraincisional unicortical femoral pins’ insertion. Supplementary material is available at https://www.sicot-j.org/10.1051/sicotj/2025001/olm.
